# Phylogenetic diversification of *glycogen synthase kinase 3/SHAGGY-like kinase *genes in plants

**DOI:** 10.1186/1471-2229-6-3

**Published:** 2006-02-21

**Authors:** Mi-Jeong Yoo, Victor A Albert, Pamela S Soltis, Douglas E Soltis

**Affiliations:** 1 Department of Botany, University of Florida, Gainesville, FL 32611, USA; 2 The Natural History Museums and Botanical Garden, University of Oslo, P. O. Box 1172 Blindern, NO-0318 Oslo, Norway; 3 Florida Museum of Natural History and the Genetics Institute, University of Florida, Gainesville, FL 32611, USA; 4 Department of Botany and the Genetics Institute, University of Florida, Gainesville, FL 32611, USA

## Abstract

**Background:**

The glycogen synthase kinase 3 (GSK3)/SHAGGY-like kinases (GSKs) are non-receptor serine/threonine protein kinases that are involved in a variety of biological processes. In contrast to the two members of the *GSK3 *family in mammals, plants appear to have a much larger set of divergent *GSK *genes. Plant GSKs are encoded by a multigene family; analysis of the *Arabidopsis *genome revealed the existence of 10 *GSK *genes that fall into four major groups. Here we characterized the structure of *Arabidopsis *and rice *GSK *genes and conducted the first broad phylogenetic analysis of the plant *GSK *gene family, covering a taxonomically diverse array of algal and land plant sequences.

**Results:**

We found that the structure of *GSK *genes is generally conserved in *Arabidopsis *and rice, although we documented examples of exon expansion and intron loss. Our phylogenetic analyses of 139 sequences revealed four major clades of *GSK *genes that correspond to the four subgroups initially recognized in *Arabidopsis*. ESTs from basal angiosperms were represented in all four major clades; *GSK *homologs from the basal angiosperm *Persea americana *(avocado) appeared in all four clades. Gymnosperm sequences occurred in clades I, III, and IV, and a sequence of the red alga *Porphyra *was sister to all green plant sequences.

**Conclusion:**

Our results indicate that (1) the plant-specific *GSK *gene lineage was established early in the history of green plants, (2) plant *GSKs *began to diversify prior to the origin of extant seed plants, (3) three of the four major clades of *GSKs *present in *Arabidopsis *and rice were established early in the evolutionary history of extant seed plants, and (4) diversification into four major clades (as initially reported in *Arabidopsis*) occurred either just prior to the origin of the angiosperms or very early in angiosperm history.

## Background

The glycogen synthase kinase 3 (GSK3)/SHAGGY-like kinases are non-receptor serine/threonine protein kinases that are involved in a variety of signal transduction pathways [1]. In animals, they are involved in cell fate determination, in metazoan pattern formation, and in tumorigenesis [2-6]. In mammals, two enzymes, GSK3α and GSK3β, are involved in the regulation of glycogen metabolism [7], in stability of the cytoskeleton [8], and in numerous processes related to oncogenesis [9]. In *Saccharomyces cerevisiae*, the *GSK3 *homologs *MCK1 *and *MDS1 *play a role in chromosomal segregation [10], and in *Schizosaccharomyces pombe *the *GSK3 *homolog *Skp1 *regulates cytokinesis [11].

In contrast to the two members of the *GSK3 *family found in mammals, plants appear to have a much larger set of divergent *GSK3/SHAGGY-like kinase *genes [12-28], with functions as numerous as in animals. Genetic and biochemical approaches indicate that different plant GSKs are involved in diverse processes, including signaling, development, and stress response. For example, the *Arabidopsis *SHAGGY-like protein kinase AtGSK1 complements the salt-sensitive phenotype of yeast calcineurin mutants [24]. In *Medicago sativa*, *GSK3 *(*WIG*) is activated by wounding [19]. *Arabidopsis AtSK11 *and *AtSK12 *participate in the regulation of flower patterning at several developmental stages [16]; both genes are expressed during perianth and gynoecium development. Cloning of the *BIN2 *(brassinosteroid-insensitive 2) locus, which is identical to *UCU1 *(*ULTRACURVATA1*) and *DWF12 *(*DWARF12*), revealed that *ASKη *(*AtSK21*) is involved in brassinosteroid signaling [25-28]. However, in contrast to the known functions of *GSK *in animals, much less is known about the specific functions of these genes in plants.

Plant GSK3/SHAGGY-like kinases are encoded by a multigene family [12-28];

*Arabidopsis *has ten different *GSK *genes [13,15-17,20,21,23]. The protein sequences of family members are highly conserved throughout the kinase domain. In contrast, the N- and C-terminal regions of the plant *GSK *genes are highly variable, consistent with observations that the various plant genes are involved in divergent biological processes. However, because the functional analyses of the plant *GSK *genes are based on mutant phenotypes or transcript expression levels [12-28], more precise analyses of mutant phenotypes without the N- and/or C-terminal regions are needed to determine whether the variable N- and C-terminal regions are related to the functional differences of plant *GSK *genes. Based on phylogenetic analyses of amino acid and cDNA sequences, *Arabidopsis GSK *genes have been grouped into four classes (I-IV) [13,15-17,21].

Besides *Arabidopsis GSK*s, *GSK3/SHAGGY-like kinase *genes have been reported from the angiosperms *Oryza sativa*, *Brassica napus*, *Medicago sativa*, *Petunia hybrida*, *Nicotiana tabacum*, and *Ricinus communis *[14,15,18,19,22,23,29,30], all of which are highly derived monocot or eudicot species. No basal eudicots or basal angiosperm lineages, representing phylogenetically ancient groups, were included in any previous analyses. Furthermore, no phylogenetic analyses of plant *GSK *genes have included sequences from diverse green plant lineages. Thus, it is not clear when plant-specific *GSK3/SHAGGY-like kinases *diverged or what complement of *GSK *genes is present in basal angiosperms or indeed other land plants. Recently, the Floral Genome Project (FGP) research consortium [31] provided expressed sequence tag (EST) sequences of *GSK *genes for a number of basal angiosperms, including *Amborella trichopoda *and the water lily *Nuphar advena *[32]. These taxa are phylogenetically important because they represent the earliest-diverging lineages of extant flowering plants [e.g., [33-42]].

In this study we examined the diversification of the *GSK3/SHAGGY-like kinase *genes in plants. Specifically, we (1) compared the structure of *GSK3/SHAGGY-like kinase *genes in *Arabidopsis *and rice, and (2) addressed whether the diversity of *GSK *genes in *Arabidopsis *is unique to *Arabidopsis *or is more generally true of all angiosperms and all land plants. For example, if the diversification of the gene family predated or coincided with the origin of the angiosperms, then ESTs from basal angiosperm taxa should appear in all major clades identified in *Arabidopsis*. Likewise, if *GSK *gene diversity in plants is ancient, basal lineages of land plants, such as mosses, should also contain orthologs to the *Arabidopsis *genes. Alternatively, some gene lineages may have diversified since the origin of the angiosperms, or land plants, and will not contain sequences from all basal lineages.

## Results and discussion

### Gene structure and patterns of sequence evolution

The structure of five *Arabidopsis GSK3/SHAGGY-like kinase *genes was reported by Dornelas et al. [15]. We sought to obtain a more comprehensive view of the structure of these genes. To accomplish this, we used the complete genome sequences now available for *Arabidopsis *and rice [43,44]; we describe the gene structure of additional *GSKs *from *Arabidopsis*, as well as the structure of *GSK*s reported from rice. We followed the numbering scheme of Dornelas et al. [15] for numbering exons and introns.

**Figure 1:**
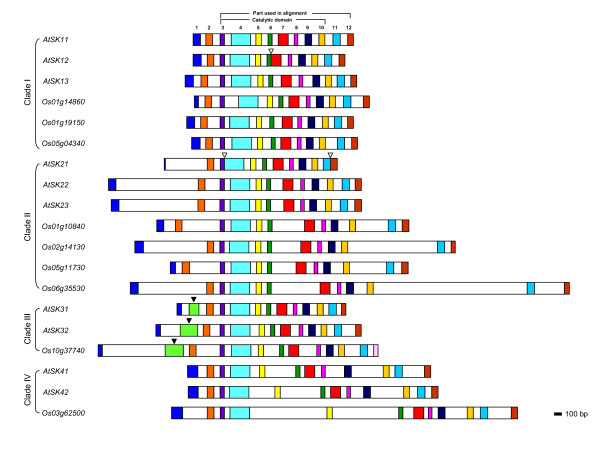
The gene structure of ten *Arabidopsis *and nine rice *GSK3/SHAGGY-like kinase *genes. The positions of the introns within the coding region are mostly conserved among *Arabidopsis *and rice *GSK *genes, except *AtSK12*, *AtSK21*, *AtSK31*, *AtSK32*, and *Os10g37740*, which either lack an intron or an exon or have additional exons. Open triangles indicate the absence of an intron. Closed triangles indicate additional exons. Boxes of identical color among sequences represent exons of the same approximate size and relative position.

The structure of *GSK *genes in *Arabidopsis *and rice is highly conserved (Figure 1). This conservation of gene structure is also apparent by inspection of the aligned sequences across a diverse array of plants, including angiosperms, gymnosperms, a fern, a moss, and green and red algae [45].

Most of the *GSK *genes have 12 exons interrupted by 11 introns, but there are some exceptions. *AtSK12 *does not contain intron 6, and *AtSK21 *does not possess introns 3 and 11. As a result, these two genes have the smallest number of exons among the *GSKs *we examined. In addition, *AtSK31 *and *AtSK32 *have one additional exon (located between exons 1 and 2) compared to most other members of the *GSK *gene family. In our phylogenetic analyses, these two genes from *Arabidopsis *appear together in a clade with a sequence from *Oryza *(*Os10g37740*), which also has one additional exon similarly located between exons 1 and 2. These results suggest that the presence of this additional exon in *Arabidopsis *and rice was inherited from a common ancestor, prior to the divergence of monocots and eudicots, suggesting that the addition of this exon was an ancient event that occurred early in the diversification of flowering plants or possibly prior to the origin of flowering plants. It would be interesting to determine whether other sequences from clade III (see phylogenetic results below) similarly have an extra exon. Tichtinsky et al. [23] reported that *PSK6.2 *and *PSK7 *from *Petunia hybrida *also have an additional exon between exons 1 and 2. However, genomic sequences are not available for other members of clade III. Recent studies demonstrate that the structure of three *GSK *genes from the moss *Physcomitrella patens *is very similar to that of *Arabidopsis *and rice [46].

The structurally variable 5' region of plant *GSKs *is composed of exons 1 and 2, and the catalytic domain is encoded by exons 3–10 [47]. The structurally variable 3' region typically comprises exons 11 and 12 (Figure 1).

The length of the *GSK *genes in *Arabidopsis *ranges from 2135 bp (*AtSK12*) to 3558 bp (*AtSK22*), whereas the length ranges from 2341 bp (*Os05g04340*) to 6186 bp (*Os06g35530*) in rice. The large variation of gene length in rice is due to the presence of long introns (up to 2173 bp in *Os06g35530*) in some genes.

### Sequence analyses

**Figure 2:**
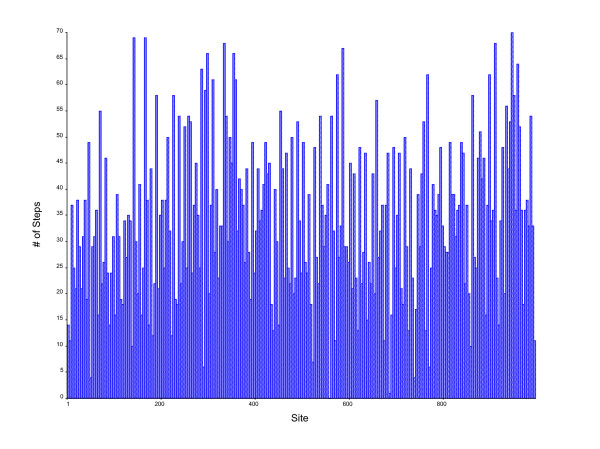
Pattern of nucleotide substitution in the coding regions of the plant *GSK *homologs based on the comparison of 116 sequences. The *x*-axis (site) was constructed based on 4-bp intervals.

**Figure 3:**
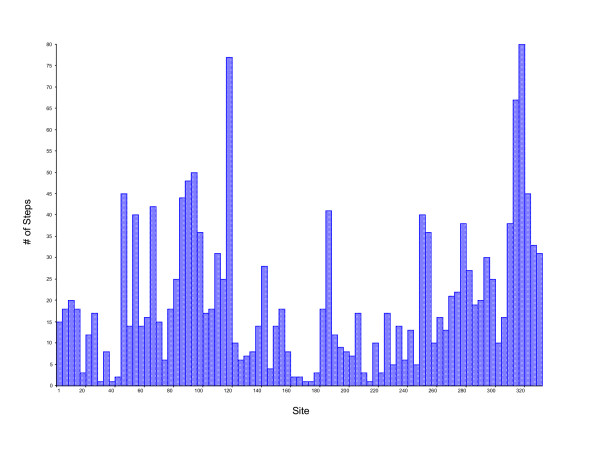
Pattern of amino acid substitution in the coding regions of the plant GSK proteins based on the comparison of 116 sequences. The *x*-axis (site) was constructed based on 4-aa intervals.

We investigated the patterns of nucleotide substitution across 116 plant-specific *GSK *homologs. This comparison provides a minimum estimate of change in a 4-position window. The substitution pattern of plant *GSK *homologs varied across the nucleotide sequences (Figure 2). The most variable 4-nucleotide window occurs at positions 945–948, with 70 substitutions in this interval. The substitution pattern of plant-specific *GSK *homologs when analyzed across amino acid sequences revealed a pattern similar to that found for nucleotide sequences. Variable regions are spread across the protein, but the most highly variable regions occur at amino acid positions 121–124 and 317–320 (Figure 3). The latter region (corresponding to the variable region of exon 12) accumulated 147 amino acid substitutions over an 8-aa interval, in a region that underwent 325 nucleotide substitutions. This high ratio of amino acid to nucleotide substitutions implies that many amino acid substitutions are tolerated in the 3' region outside of the catalytic domain (Figure 3). In contrast, amino acid positions 29–32, 37–44, and 161–180 were conserved, although these regions were not conserved at the nucleotide level, suggesting that selection and/or functional constraints may be important in this part of the protein.

**Figure 4:**
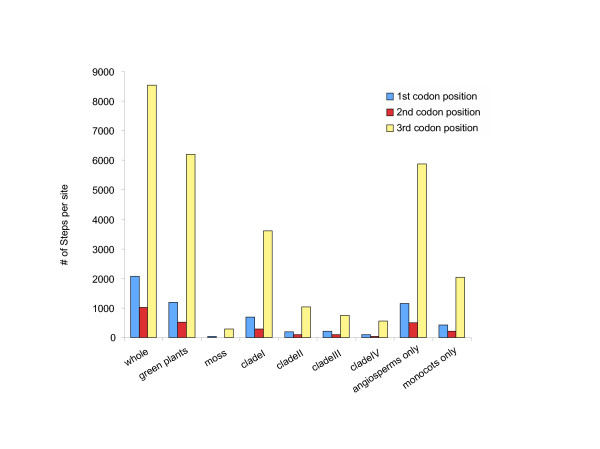
Mean number of inferred nucleotide substitutions by codon position based on the comparison of 139 *GSK *homologs. Subsets of the full data set are based on the results of the phylogenetic analysis of *GSK*s (green plants, moss, clade I, clade II, clade III, clade IV) or represent well-recognized organismal groups (angiosperms, monocots).

Changes at the first, second, and third codon positions varied substantially. Substitutions in third positions were much more frequent than those at first and second positions (Figure 4). The ratio of base substitutions by codon position is 2.0: 1.0: 7.6. A similar pattern was observed in each clade analyzed: green plants, mosses, clade I, II, III, and IV. Substitutions also vary similarly among organismal groups, regardless of gene clade, for example, among all angiosperm sequences and among all monocot sequences (Figure 4). This result implies a similar pattern of base substitution in diverse gene lineages and organismal lineages.

### Phylogeny of GSK3/SHAGGY-like kinase genes

A total of 842 variable sites was found in the nucleotide sequences, 735 of which were parsimony-informative. Seventeen most parsimonious trees with a length of 11641 steps were obtained from the maximum parsimony (MP) analysis. The consistency index (CI) was 0.1522, and the retention index (RI) was 0.5789. In the amino acid analysis, 288 variable sites were detected, with 234 parsimony-informative; 77 most parsimonious trees of 2156 steps were obtained (CI = 0.4935; RI = 0.7532).

**Figure 5:**
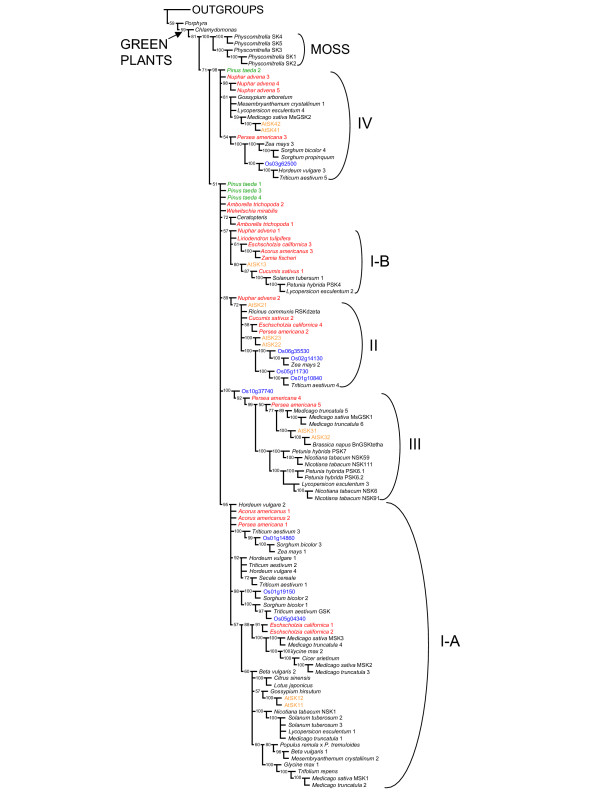
Phylogenetic tree resulting from analysis of nucleotides using Support Weighting with jackknife values from non-weighted analysis. Orange labels indicate *GSK *homologs from *Arabidopsis*, and blue labels designate rice sequences. *GSK *homologs from FGP ESTs are labeled in red. *Pinus *ESTs are labeled in green.

**Figure 6:**
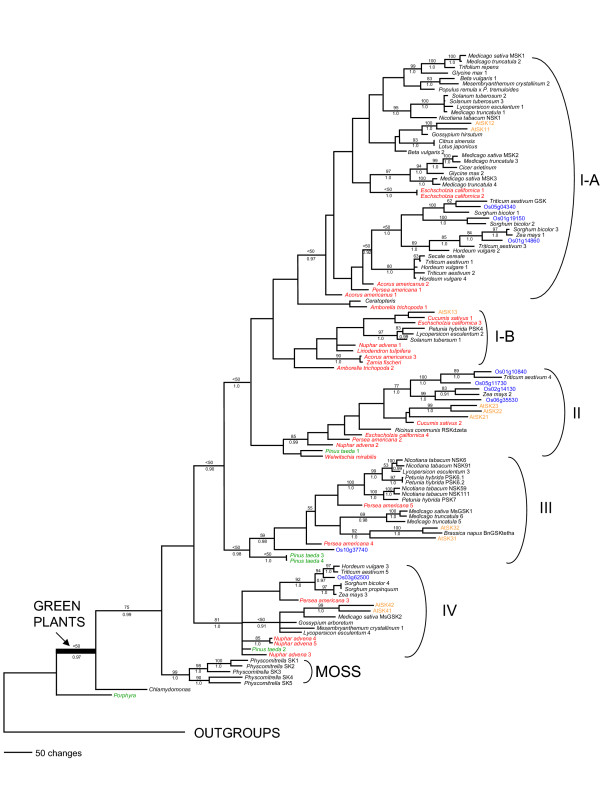
Strict consensus tree of 17 most parsimonious trees (length = 11641; CI = 0.1522; RI = 0.5789) of *GSK3/SHAGGY-like kinase *homologs from plants, animals, protists, and fungi based on sequence alignment of the 1044 nucleotides encoding the catalytic domain and part of the 3' end of the sequences. Numbers above the branches are bootstrap values; only values over 50% are indicated. Numbers below the branches are posterior probabilities from the Bayesian analysis; only values over 0.90 are indicated. Orange labels indicate *GSK *homologs from *Arabidopsis*, and blue labels designate rice sequences. *GSK *homologs from FGP ESTs are labeled in red. *Pinus *ESTs are labeled in green.

**Figure 7:**
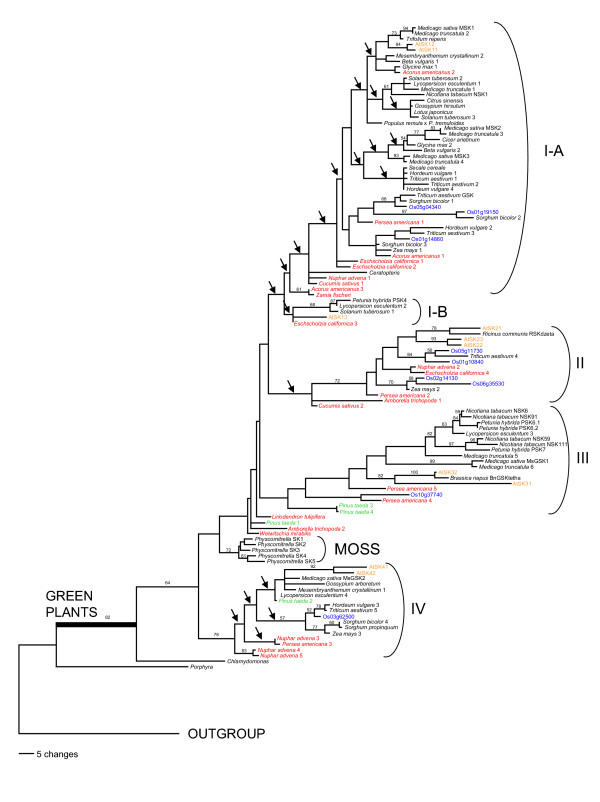
50% majority rule consensus tree of 77 most parsimonious trees (length = 2156; CI = 0.4935; RI = 0.7532) of GSK3/SHAGGY-like kinase proteins from plants, animals, protists, and fungi based on sequence alignment of the 348 amino acids of the catalytic domain and part of the C terminus. Numbers above the branches are bootstrap values; only values over 50% are indicated. Arrows on the nodes indicated the collapsed braches in the strict consensus tree. Orange labels indicate GSK proteins from *Arabidopsis*, and blue labels designate rice proteins. GSK proteins inferred from FGP ESTs are labeled in red, and those from *Pinus *ESTs are labeled in green.

The clades identified in the support-weighted tree based on nucleotide sequences (SW; Figure 5) are very similar to those of the maximum parsimony tree based on the same data set (MP-N; Figure 6), although relationships among basal nodes are not resolved in the support-weighted tree. Furthermore, the clades found in the trees based on nucleotide sequences (both MP and SW) are very similar to those found in the MP trees based on translated amino acid sequences (MP-AA; Figure 7). Therefore, AT content, codon usage, and other molecular evolutionary biases do not appear to have compromised the reliability of the nucleotide-based results. In fact, the nucleotide data are more informative than the amino acid sequences, yielding greater support for most clades (see Figures 5, 6, 7). However, support for most clades is quite low in all analyses.

The clades found in the Bayesian phylogenetic analysis based on nucleotide sequences are almost identical to those of the maximum parsimony tree based on the same data set. Therefore, the posterior probabilities are indicated on the maximum parsimony strict consensus tree (MP-N) (Figure 6).

In all four phylogenetic analyses, all of the land plant *GSK *sequences formed a clade distinct from non-plant sequences with high values of internal support as measured by bootstrap, posterior probabilities, and jackknife resamplings (Figures 5, 6, 7). In all four analyses, the *Porphyra *sequence is sister to all green plant sequences (0.97 posterior probability, support values of 59%, <50%, and 82% from parsimony jackknifing mapped onto the SW tree, MP-N, and MP-AA, respectively), and the *Chlamydomonas *sequence is sister to all other green plant *GSK*s (0.99 posterior probability, support values of 81%, 75%, and 64% from parsimony jackknifing mapped onto the SW tree, MP-N, and MP-AA, respectively).

The trees from all four analyses recovered five major clades of sequences within land plants. One clade is composed only of sequences from the moss *Physcomitrella *(1.0 posterior probability, support values of 100%, 99%, and 72% from parsimony jackknifing mapped onto the SW tree, MP-N, and MP-AA, respectively), and the remaining four clades (I, II, III, and IV) correspond to the *GSK *subgroups recognized in *Arabidopsis *[13,15-17,21]. Relationships among these five clades varied among the analyses, but internal support was weak except in the Bayesian analysis. A large clade containing clades I, II, and III received a posterior probability of 0.90, and a clade including clades I and II had a posterior probability of 1.0 (Figure 6).

The MP-N tree (Figure 6) shows the moss clade as sister to the remaining four clades, whereas the MP-AA tree places the moss clade as sister to clades I, II, and III, with clade IV sister to this entire clade of moss + clades I, II, and III (Figure 7). The SW analysis also placed the moss clade as sister to the remaining four clades, and clade I was split into two separate clades (Figure 5). The fact that several taxa bear multiple *GSK*s that fall into separate subclades within clade I suggests that "clade I" may actually represent the products of an additional ancient duplication. However, the non-monophyly of clade I in the SW tree, lack of bootstrap support >50% in the MP trees, and the low posterior probability in the Bayesian analysis suggest that these two subclades may not be each other's closest relatives.

Although we recovered four major clades that correspond to the four groups recognized in *Arabidopsis *by Dornelas et al. [15], relationships among and within these clades are generally not well supported based on analyses of either nucleotide or amino acid sequences (Figure 5, 6, 7), apparently due to the conflict among characters. Low support was not due to the choice of outgroups. We repeated the phylogenetic analyses using only *Chlamydomonas *as an outgroup and obtained the same topology and similar levels of support.

Clade IV was supported most strongly, with 98% jackknife support (on the SW tree; Figure 5), 1.0 posterior probability, and 81% and 78% bootstrap support from the MP-N and MP-AA analyses. Clade III received jackknife support of 100% (SW tree), 0.98 posterior probability, and bootstrap support less than 50% in both MP analyses. Clade II was supported by a jackknife value of 89% (on the SW tree; Figure 5), 0.99 posterior probability, and bootstrap values of 85% and 72%, respectively, in the MP-N and MP-AA analyses. Clade I received less than 50% bootstrap support in both MP analyses and <0.90 posterior probability in the Bayesian analysis, and the SW analysis split this clade into two parts, with jackknife values of 57% and 95%, respectively, mapped onto the SW tree (Figure 5).

*Oryza *sequences were included in the same four major clades with the *Arabidopsis GSK*s (Figures 5, 6, 7). Clade I contains three rice sequences, *Os01g19150*, *Os01g14860*, and *Os05g04340*, and clade II includes *Os01g10840*, *Os05g11730*, *Os06g35530*, and *Os02g14130 *in all trees. The presence of duplicate *Oryza *sequences within individual clades raises the possibility that some rice *GSK *genes may have resulted from relatively recent gene duplication, as reported in *Arabidopsis *[15]. Recently reported evidence of genome duplication in rice [48] may explain, at least in part, the multiple *Oryza *sequences within clades I and II. Sequences of several other plant genera are found in three of the four clades. Sequences of the grasses *Triticum *and *Zea *are found in clades I, II, and IV, and *GSK*s from the eudicots *Medicago *and *Lycopersicon *are found in clades I, III, and IV.

Clade IV includes *AtSK41 *and *AtSK42 *from *Arabidopsis*, plus sequences from other eudicots, monocots, and the basal angiosperms *Persea americana *and *Nuphar advena*. *Nuphar advena *4 and 5 form a clade with 83% bootstrap support, appearing well separated from *Nuphar advena *3 (Figure 7). These data for *Nuphar advena *suggest at least one gene duplication in clade IV and indicate a diversity of *GSK *genes within some basal angiosperm species, comparable to that observed within the eudicot *Arabidopsis*. Finally, *Pinus taeda *2 grouped with eudicot sequences in the MP-AA analysis (Figure 7), but in other analyses it failed to form a subclade.

In clade III, two *Pinus *ESTs (*Pinus taeda *3 and 4) were sister to all other sequences in both MP trees, but this relationship was weakly supported (<50%) even though the posterior probability was high (0.98). In addition, in the SW tree, these two *Pinus *sequences failed to form a clade (Figure 5). Also within clade III, one *Persea *EST sequence is sister to a eudicot-specific clade that contains sequences from *Nicotiana*, *Petunia*, *Lycopersicon*, *Medicago*, *Brassica*, and *Arabidopsis *(*AtSK31 *and *AtSK32*).

Clade II contains the *Arabidopsis *sequences *AtSK21*, *AtSK22*, and *AtSK23*. The sequences from rice, wheat, and maize formed a clade with 77% bootstrap support in the MP-N analysis, 1.0 posterior probability, and 100% jackknife support mapped on the SW tree; this clade was not recovered in the MP-AA analysis. This clade also includes sequences from the basal angiosperms *Persea *and *Nuphar *in all trees and from *Amborella *in the MP-AA tree. Sequences from the eudicots *Eschscholzia*, *Ricinus*, and *Cucumis *are also included in clade II.

Clade I contains the *Arabidopsis *sequences *AtSK11 *and *AtSK12*, which formed a sister pair in all analyses (Figures 5, 6, 7); *AtSK13 *appeared in a separate subclade near the base of clade I, well removed from *AtSK11 *and *AtSK12 *in the MP-N and MP-AA trees. In the SW tree, clade I is not monophyletic, and these sequences fall into two clades (I-A and I-B), with 52% and 93% jackknife support, respectively, mapped on the SW tree. *AtSK11 *and *AtSK12 *occur in I-A, and *AtSK13 *occurs in I-B (Figure 5). Expression studies have demonstrated that both *AtSK11 *and *AtSK12 *seem to be involved in flower development [13,16]. In contrast, *AtSK13 *plays a role in the response to saline treatment and osmotic pressure. It is therefore not surprising that *AtSK11 *and *AtSK12 *are not closely related to *AtSK13*, although phylogenetic position and function are not always coupled. Clade I also contains multiple copies of *GSK*s from the basal monocot *Acorus*, two in I-A and one in I-B in the SW tree. The relationship between the two sequences in I-A is not resolved, and their positions in the two MP consensus trees did not receive bootstrap support >50%; it is therefore possible that these two sequences are in fact sisters and represent the product of a gene duplication within the *Acorus *lineage. The functions of these divergent copies remain to be investigated.

From an evolutionary standpoint, it is significant that ESTs from basal angiosperms were represented in all four major clades in all analyses (Figures 5, 6, 7). ESTs of *Nuphar *(Nymphaeaceae) occur in three of the four clades (Figures 5, 6, 7). ESTs of *Amborella*, the sister to all other living flowering plants (either alone or with Nymphaeales; reviewed in [49]), are found in clades I and II. ESTs of *Persea*, the avocado (Lauraceae), occur in clades I, II, III, and IV, and an EST of *Liriodendron *(tulip poplar; Magnoliaceae) is in clade I. ESTs of *Eschscholzia *(poppy; Paveraceae), a basal eudicot, are in clades I and II. Sequences from the basal angiosperm lineages typically attach at, or near, the base of the clades in which they appear. For example, a sequence of *Nuphar *is sister to other sequences in clade IV in the MP-AA tree. A sequence of *Amborella *attaches near the base of clade I in the MP-N tree and clade II in the MP-AA tree, and a sequence of *Persea *attaches very close to the base of clades II and III in the MP-N and MP-AA tree.

There is a distinct monocot subclade in both clades II and IV, and most of the monocots form two or three subclades in clade I. These monocot-specific subclades are particularly evident in the MP-N tree (Figure 6). Within most clades, the eudicot sequences form a distinct subclade, for example, the subclade of *Nicotiana*, *Petunia*, *Lycopersicon*, and *Medicago *sequences within clade III. In clade II the *GSK *homologs of the eudicots *Arabidopsis *and *Ricinus *form a subclade. The other eudicot member of clade II, *Cucumis*, does not appear with the *Arabidopsis *and *Ricinus *sequences. However, the *Cucumis *sequence is a partial sequence (only 72 amino acid residues), which could affect its phylogenetic placement. Recently, Wiens [50,51] reviewed the effect of missing data in phylogenetic analyses, and his simulations showed that incomplete sequences can be accurately placed in phylogenies; furthermore, they typically do not impact the overall tree, in agreement with empirical studies [e.g., [39,40]]. We analyzed our data set with and without the partial *Cucumis *sequence, but removal of this sequence did not alter the topology of the remaining sequences.

Sequences of *GSK3/SHAGGY-like kinases *are also available for a fern and for several gymnosperms. An EST of the fern *Ceratopteris *appeared within clade I, as sister to a subclade that includes *AtSK11 *and *AtSK12 *in the MP-AA tree (Figure 7). Sequences from *Zamia *attached near the base of clade I, a sequence of *Welwitschia *was sister to clades I, II, and III, and the four EST sequences of *Pinus taeda *appeared in clades III and IV (MP-AA), although these positions varied in the SW and MP-N trees (Figures 5, 6). The placement of gymnosperm sequences in clades I, III, and IV in the MP-AA tree suggests that *GSK*s diversified to some extent prior to the origin of seed plants, over 300 million years ago [e.g., [52,53]]. In addition, the presence of a *GSK *sequence in *Porphyra *and its phylogenetic placement as sister to all green plant sequences (at least in the two MP analyses) indicates that the plant-specific *GSK*s were already established before the origin of green plants, the oldest fossils of which are unicellular and filamentous green algae from the Neoproterozoic of Australia (900 mya; [54,55]) and Spitzbergen (700–800 mya; [56,57]; reviewed in [52]). Taken together, our structural and phylogenetic analyses indicate that plant *GSK3/shaggy-like kinases *were established prior to, or at least early in, the diversification of green plants and that the common ancestor of seed plants already had a diverse tool kit of *GSK3/shaggy-like kinase *genes that could be used for various signaling-related processes. Future comparative studies of gene function, based on orthologous genes, may be informative about patterns of functional diversification of *GSK *genes.

## Conclusion

The structure of *GSK *genes in *Arabidopsis *and rice is highly conserved, and most *GSK *genes have 12 exons interrupted by 11 introns. Genes included in the same clade based on parsimony analyses share similar structural characteristics. Our phylogenetic results indicate that the plant-specific *GSK *gene lineage was established prior to, or early in, the history of green plants, and plant *GSK*s began to diversify prior to the origin of extant seed plants. In addition, at least three of the four major clades of *GSK*s (I, III, IV) present in *Arabidopsis *and rice were established early in the history of extant seed plants. Sequences of basal angiosperms are present in all four of the major *GSK *clades, indicating that the fourth major subgroup of these genes (II) was established either early in angiosperm evolution or prior to the origin of the angiosperms (but after their last common ancestor with extant gymnosperms), if the absence of Clade II sequences from gymnosperms is real and not an artifact of limited sampling. In addition, our data indicate that *GSK *gene duplication events may have occurred in several of the basal angiosperms investigated, most notably *Nuphar*. Thus, duplication of *GSK *genes, which is prevalent in both *Arabidopsis *and rice, has also occurred in basal angiosperms. This phylogenetic analysis of numerous plant *GSK *sequences provides a framework for the investigation of the functional genetics of *GSK*s in signaling, development, and stress response.

## Methods

### Data retrieval

A search for *GSK3/SHAGGY-like kinase *homologs was performed using BLAST [58,59] at the websites of NCBI [60], TIGR [61], PlantGDB [62], Kazusa DNA Research Institute [63], and the FGP [31]. We started our search with 10 *Arabidopsis *and nine rice sequences, and then continued with various published *GSK3/SHAGGY-like kinase *homologs from human, yeast, *Drosophila*, *Brassica*, *Medicago*, *Petunia*, *Nicotiana*, and *Ricinus *to identify as many *GSK *homologs as possible from protists, fungi, animals, and plants. Putative *GSK *homology was defined initially by sequence similarity when the sequences were retrieved and then confirmed by phylogenetic analysis (see below). A total of 139 *GSK *homologs was collected, of which 73 sequences were ESTs: 26 ESTs from 10 taxa at the FGP web site, 40 ESTs from 17 taxa at the PlantGDB web site, 5 ESTs from the NCBI web site (*Ceratopteris *and *Pinus*), and two ESTs from the Kazusa DNA Research Institute database (*Clamydomonas *and *Porphyra*). Some ESTs were integrated into a contig, which was constructed using the CAP3 Sequence Assembly Program [64], and therefore some gene designations have several accession numbers (Additional File 1). Of the remaining 66 sequences, 43 were previously reported land plant sequences, and 23 were sequences from protists, fungi, and animals (Additional File 1).

### Sequence alignment

All sequences were translated into amino acid sequences using Se-Al [65]. The sequences corresponding to the catalytic domain (as defined by Hanks [47]; 285 amino acid residues corresponding to exon 3 to exon 10 in *Arabidopsis*; see Figure 1) and part of the 3' region (corresponding to 78 amino acid residues; exons 11 and 12 in *Arabidopsis*) were aligned manually in a stepwise manner using Se-Al; other regions were too variable to align. The aligned matrix therefore comprised exons 3 to 12 and was 348 amino acid residues in length; the average length of all included sequences was 293 amino acid residues, and the average length of the translated EST sequences was 193 amino acid residues. The aligned sequences were exported for phylogenetic analyses as separate data matrices of nucleotide sequences and amino acid sequences, and all data matrices and trees were deposited in TreeBASE (Study accession S1459, matrix accessions M2623-M2624) [45]. For *Arabidopsis *and rice, the genomic sequences were aligned and compared with cDNA sequences to investigate gene structure.

### Sequence analyses

A series of analyses was conducted to explore the pattern of sequence evolution in *GSK *homologs. We investigated patterns of substitution across both nucleotide and protein sequences using the CHART option of MacClade 4.05 [66], using 116 plant-specific *GSK *homologs and Tree 1, selected arbitrarily from the phylogenetic analysis. This approach provides a minimum estimate of change for each site. Plotting of substitutions was conducted across a 4-bp or 4-amino acid interval on the x-axis. The analyses were conducted across the entire aligned sequences. We tested for variation in mean substitution rate among codon positions using the CHART option of MacClade 4.05, across the entire data set, within all green plants, within mosses, and within each of the four major clades of seed plant sequences identified by phylogenetic analyses.

### Phylogenetic analyses

Maximum parsimony analyses were conducted with (i) equally weighted characters and character states and (ii) support weighting [67]. Equally weighted parsimony analyses for matrices of nucleotides and amino acids were conducted using PAUP* 4.0b10 [68]. The search strategy involved 100 random addition replicates with TBR branch swapping, saving all optimal trees. Gaps were treated as missing data. To assess support for each node, bootstrap analyses [69] were performed using 100 replicate heuristic searches, each with 10 random addition replicates and TBR branch swapping, saving all optimal trees.

The support weighting method [67] provides an alternative approach to assessing internal support for phylogenetic results, by measuring the degree to which changes in a character (site) are concentrated in the supported branches of a tree. Jackknife resampling was used to generate randomly selected suites of initial weights for successive support weighting, providing a means of assessing the stability of branches supported in a standard parsimony jackknife tree [67,70]. We applied the support weighting method to the nucleotide data matrix. Support values mapped onto the support-weighted tree topology were generated by standard parsimony jackknifing [70] of the original data matrix using 1000 replicates with SPR branch swapping on each of 10 random data entry orders.

A Bayesian phylogenetic analysis was performed using MrBayes 3.1.1 [71] to compare the tree topology and support values to those obtained from maximum parsimony analyses. The GTR + I + Γ model was selected by the Akaike information criterion (AIC) in ModelTest v.3.6 [72,73] and applied for the Bayesian analysis. Default parameter values were used for the priors. The analysis was run for 20 million generations, sampling trees every 1000 generations. The first 3000 trees produced during 3 million generations were discarded as burn-in, and the 50% majority-rule consensus of the remaining trees was used to obtain posterior probabilities. Two chains were run, and results from both chains were combined as convergence diagnostics indicated they had converged on similar results (the average standard deviation of split frequencies at 20 million generations was 0.062054).

In previous phylogenetic analyses [13], *mitogen activated protein kinase *(*MAPK*) and *cyclin-dependent kinase *(*CDK*) sequences were shown to be the sister group to a clade of all *GSK *homologs. We analyzed plant *MAPK*/*CDK*/*Casein kinase II*/*GSK *sequences because these four kinases are included in the same group [74]. In an unrooted tree, *GSK *sequences formed a clade in which non-plant *GSK *homologs were sister to plant *GSK*s (tree not shown). As a result, we used non-plant *GSK*s as outgroups for analysis of all plant-specific *GSK *homologs.

## Authors' contributions

M-JY carried out the sequence analysis, the equally weighted maximum parsimony analysis, and the Bayesian analysis, and with PSS and DES wrote the manuscript. VAA performed the support weighting analysis. PSS and DES supervised the project. All authors read and approved the final submission.

## Supplementary Material

Additional File 1 List of GSK3/SHAGGY-like kinase homologs used in this study. Some gene designations represent contigs constructed from multiple sequences and therefore have several accession numbers. Click here for file
